# Demographic and Histological Predictors of Survival in Patients With Gastric and Esophageal Carcinoma

**DOI:** 10.5812/ircmj.11847

**Published:** 2013-07-05

**Authors:** Yousef Veisani, Ali Delpisheh, Kourosh Sayehmiri, Ezzatollah Rahimi

**Affiliations:** 1 Students' Research Committee, Ilam University of Medical Sciences, Ilam, IR Iran; 2 Department of Clinical Epidemiology, Ilam University of Medical Sciences, Ilam, IR Iran; 3 Prevention of Psychosocial Injuries Department, Research Centre, Ilam, IR Iran; 4 Liver and Digestive Research Centre, Kurdistan University of Medical Sciences, Sanandaj, IR Iran

**Keywords:** Gastric Cancer, Esophageal Cancer, Histological Factor, Demographic Factor, Survival

## Abstract

**Background:**

Little is known about the possible influence of demographic and histological risk factors on the survival of patients with esophageal and gastric cancer.

**Objectives:**

Based on the available registry and follow-up information, this study compares 1-5 year survival rate of gastric and esophageal cancer.

**Patients and Methods:**

Through a concurrent (prospective) cohort study, all 366 patients with definite diagnosis of esophageal and gastric cancer who had been hospitalized at the Towhid Hospital, Sanandaj city, Kurdistan province, western Iran during a five-year period from 2006 onwards were recruited. The survival time of patients stratified by this grouping method were analyzed by Kaplan-Meier analysis and Cox regression.

**Results:**

Amongst the 366 patients, 23 had esophageal adenocarcinoma, 94 esophageal squamous-cell carcinoma and 239 had gastric adenocarcinoma. Age at diagnosis (P = < 0.001), tumor grade (P = 0.008) and practice treatment (P = < 0.001) had significant associations with the variation of survival rates in patients with esophageal but not with gastric cancer. The five-year survival rates (by year) for esophageal cancer were 49%, 27%, 24%, 22% and 19%, respectively and for gastric cancer were 41%, 17%, 13%, 10% and 5.4%, respectively.

**Conclusions:**

Major differences between these cancers were seen in the survival rates of patients and their incidence classified by sex. Age at diagnosis and histological types were prognostic factors for survival of patients with esophageal cancer but this wasn’t the case for gastric cancer.

## 1. Background

Gastric cancer remains to be a major cause of cancer mortality. Since the last decades, gastric cancer mortality rate has decreased globally (1). However, it still remains a major public health concern with a poor prognosis and a high mortality (2). In contrast, the incidence of esophageal cancer is increasing (3). In the United States, the incidence of esophageal cancer has had a six-fold increase caused by the rise in the incidence of esophageal adenocarcinoma (4). In Europe there has also been an increase in the incidence of esophageal adenocarcinoma in men, but not in women (5). A similar pattern has already been found in Iran (6). The disease is highly lethal, with an overall five-year survival rate of less than 10%. The high mortality is due to the late onset of symptoms (7). In the Ardabil province, during 2007, one and five year survival rates of patients with upper gastrointestinal cancer were 40.5% and 0.8% respectively (8). Geographic variation and temporal trends in the epidemiology of esophageal and gastric cancers vary according to tumor morphology and organ subsite. Geographic variations in Iran show that the incidence and mortality of upper GI (Gastrointestinal) cancers is higher in the West and North West regions and in the Kurdistan province in particular (9). 

## 2. Objectives

Based on the available registry and follow-up information, this study compares 1-5 year survival rates of gastric and esophageal cancers.

## 3. Patients and Methods

### 3.1. Source of Data

Data were sourced mainly through patient reports from pathology laboratories and hospital database records. Through a concurrent (prospective) study using the censes method; all eligible patients with upper GI cancers (134 esophagus, and 249 gastric cancer) who had been hospitalized at the Towhid Hospital, Sanandaj city, Kurdistan province western Iran were recruited. Inclusion criteria were patients with definite diagnosis of upper GI cancer during a five-year period from 2006 onwards. Samples were coded under the direct supervision of clinical pathologists according to the International Classification of Diseases for Oncology (10). Clinical data such as practice treatments were obtained through a structured questionnaire and the patients’ clinical records. Vital status and date of death were determined through the official death certificates, with a maximum follow-up of 90 months. Survival time (in months) was calculated from the date of diagnosis to the date of death or last follow-up. A failure was defined as death by any cause during the follow-up period and patients alive at the end of the follow-up period were censored. Overall, 17 patients were excluded from the analyses according to the exclusion criteria (8 patients were lost to follow-up, 9 had illegible data, and 3 patients migrated). Overall, 366 (127 esophagus, and 239 gastric cancer) patients were enrolled. Clinical and pathologic variables, which were sub-layered into age, gender, setting, histological type of tumor and practice treatment were entered into parametric regression models (by considering and not considering heterogeneity) for multivariate analysis in order to assess the relationships between the characteristics and prognostic factors for survivors. The present study was approved (Code No: 91002, Date: 22.08.2012) by the Ilam University of Medical Sciences, Ethics Committee by consideration of the publication data results in general.

### 3.2. Statistical Analysis

The Kaplan Meier and Log rank statistic methods were used to compare survival rates in different subgroups. Using life table, survival rates and survival density function was assessed at one-year intervals.Breslow (Generalized wilcoxon) statistics was used to compare median survival time of three age groups. The Cox hazards regression analysis was also used to investigate the effect of the variables and adjust for the effect of demographical and pathological variables on survival. Graphical (diagram Log (S) t vs. time) and analytical (time-varying covariate) methods were applied to test the appropriateness of Cox’s proportional hazard (11). Multiple Cox regression analysis was used to identify independent predictors for patient survival using a backward stepwise approach with an entry limit of P < 0.1 and a removal limit of P < 0.05. The survival time of patients stratified by this grouping method were analyzed by the Kaplan-Meier analysis and Cox regression as described earlier. T tests, chi-square tests and Fisher’s exact test were used to compare patient characteristics and tumor factors between the populations. All statistical analyses were performed using SPSS16.0.

## 4. Results

Out of the 366 studied patients, 23 (18.1%) were diagnosed with esophageal adenocarcinoma (AC), 94 patients (74.0%) with esophageal squamous- cell carcinoma (SCC) and 239 with gastric cancer. Adenocarcinoma intestinal type was the predominant histological type of gastric tumor (129 patients; 54%). Mean age ± standard deviation (SD) at diagnosis of patients with gastric cancer was 68.8 ± 11.97 years and 65.38 ± 11.62 for esophageal cancer (P = < 0.01) (Table 1). 

**Table 1.** Characteristic of Patients With Gastric and Esophageal Cancers

Factors	Gastric Cancer, No (%)	Esophageal Cancer, No (%)
**Gender**		
Male	178 (74.5)	70 (55.1)
Female	61 (25.5)	57 (44.9)
**Age**		
45 >	9 (3.8)	11 (8.7)
46 - 65	90 (37.7)	54 (42.5)
66 <	139 (58.8)	42 (48.8)
**Setting**		
City	120 (50.2)	82 (64.6)
Village	119 (49.8)	45 (35.4)
**Histological type ** ^**a**^		
AC/Intestinal	129 (54.0)	23 (18.1)
SCC/Diffuse	84 (35.1)	94 (74.0)
Other	26 (10.9)	10 (7.9)
**Histology grade**		
Poor	28 (11.8)	16 (12.6)
Moderate	25 (10.5)	35 (27.6)
Well	31 (13.0)	42 (48.8)
No difference	154 (64.4)	14 (11.0)
**Practice treatment**		
Surgery	19 (12.7)	15 (11.8)
Chemotropic	53 (35.3)	30 (23.6)
Radiotherapy	4 (1.7)	7 (5.5)

^a^ Esophageal cancer (Adenocarcinoma type (AC), squamous- cell carcinoma type (SCC) and other (unknown type)); Gastric cancer (Intestinal type, Diffuse type and other (unknown type))

The majority of patients were male and older than 65 years for gastric cancer and 46-65 years old for esophageal cancer. The median ± (SD) survival rate for patients with esophageal cancer was 10.0 ± 1.05 months and for those with gastric cancer, this was 11 ± 0.46 months. In those with esophageal cancer, patients with the histopathology of “SCC” had the lowest survival rate (median: 7 months); other cases lived more than 13 months after the diagnosis. Selected characteristics of the patients are shown in Table 2 and Table 3. 

**Table 2.** Median Survival of Patients With Esophageal Cancer

Factors	Median Survival, (CI 95%)	P value
**Gender**		0.480
Male	10 (7.97 - 12.03)	
Female	12 (6.46 - 17.54)	
**Age**		< 0.001
45 >	25 (10.94 - 31.33)	
46 - 65	18 (10.94 - 25.06)	
66 <	6 (4.6 - 7.4)	
**Setting**		0.148
City	10 (8.05 - 11.95)	
Village	14 (10.05 - 17.95)	
**Histological type ** ^**a**^ ****		< 0.001
AC	7 (4.18 - 9.82)	
SCC	13 (9.34 - 16.66)	
Other	10 (7.93 - 12.07)	
**Histology grade**		0.008
Poor	7 (1.12 - 12.88)	
Moderate	22 (6.06 - 37.94)	
Well	9 (6.80 - 11.20)	
No difference	12 (7.41 - 16.59)	
**Practice treatment**		< 0.001
Surgery	12 (4.42 - 19.57)	
Chemotropic	5 (3.92 - 6.07)	
Radiotherapy	5 (2.91 - 7.09)	

^a^ Adenocarcinoma type (AC), squamous- cell carcinoma type (SCC) and other (unknown type)

**Table 3.** Median Survival of Patients With Gastric Cancer

Factors	Median Survival, (CI 95%)	P value
**Gender**		0.361
Male	11 (9.54 - 12.06)	
Female	13 (11.46 - 14.86)	
**Age**		0.220
45 >	12 (7.98 - 16.02)	
46 - 65	12 (8.54 - 16.46)	
66 <	10 (8.71 - 11.29)	
**Setting**		0.198
City	11 (9.47 - 12.53)	
Village	10 (10.08 - 11.92)	
**Histological type**		0.611
Intestinal	11 (9.82 - 12.18)	
diffuse	13 (11.21 - 14.79)	
Other	10 (9.19 - 10.81)	
**Histology grade**		0.309
Poor	10 (7.06 - 12.94)	
Moderate	16 (13.94 - 8.06)	
Well	13 (9.17 - 16.83)	
No difference	11 (10.09 - 11.91)	
**Practice treatment**		0.367
Surgery	12 (8.90 - 15.10)	
Chemotropic	9 (6.48 - 15.52)	
Radiotherapy	7 (4.45 - 9.55)	

**Figure 1.**
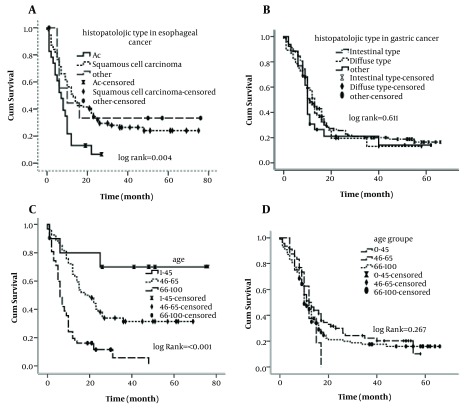
The Associations Between Demographic and Histological Factors and Survival of Esophageal and Gastric Cancer Patients, A) By Histology of Tumor in Esophageal Cancer, B) By Histology of Tumor in Gastric Cancer, C) By Age at Diagnosis in Esophageal Cancer, D) By Age at Diagnosis in Gastric Cancer

Gender and setting of patients had no significant effects on survival rate variation in univariate analysis of both cancers. Age at diagnosis (P = < 0.001), practice treatment (P = < 0.001), histology grade (P = 0.008) and tumor histology (P = 0.004) were significantly associated with survival rate variations of patients with esophageal and there was no significant association regarding gastric cancer patients (Figure 1). 

Potential prognostic factors of patients with gastric cancer showed that tumor histology, histology grade, practice treatment, gender, age, and place of residence on prognosis were not significant in a multivariate model adjusted for follow-up time. For esophageal cancer, Cox-regression analysis using demographic and histological factors and by having age of 45 > years as a reference revealed that patients whose age was 46-65 years at diagnosis (HR = 3.43, 95%, CI = 1.03 – 11.41, P = 0.044), 66 < year (HR = 9.78, 95% CI = 2.93 – 32.64, P = < 0.001) had an increased risk for disease progression and death. Cox-regression analysis for low grade as a reference revealed that patients whose tumors were moderately differentiated (HR = 0.52, 95%, CI = 0.25 - 1.07, P = 0.078) and well differentiated (HR = 0.98, 95%, CI = 0.51 - 1.85, P = 0.951) had a decreased risk for death from esophagus cancer. Cox regression coefficient (β) analysis showed that patients with tumors located at the middle (β = -0.91) and upper region of the esophagus (β = -0.13) had lower death rates compared to those with tumors located at the lower part of the esophagus. Similar results were obtained for tumor grades (Table 4). 

**Table 4.** Multivariate Cox Regression Analysis for Patients With Esophageal Cancer

Characteristics	β	HR (95% CI)	P value
**Age**	-	overall	< 0.001
45 >	reference	1	reference
46 - 65	1.23	3.43 (1.03-11.41)	0.044
< 65	2.28	9.78 (2.93-32.64)	< 0.001
**Histology grade**	-	overall	0.085
Poor	reference	1	reference
Moderate	-0.64	0.52 (0.25-1.07)	0.078
Well	-0.02	0.98 (0.51-1.85)	0.951
**Location of tumor**	-	overall	0.009
Lower	reference	1	reference
Middle	-0.91	0.40 (0.22-0.73)	0.003
Upper	-0.13	0.53 (0.33-0.85)	0.009

**Figure 2.**
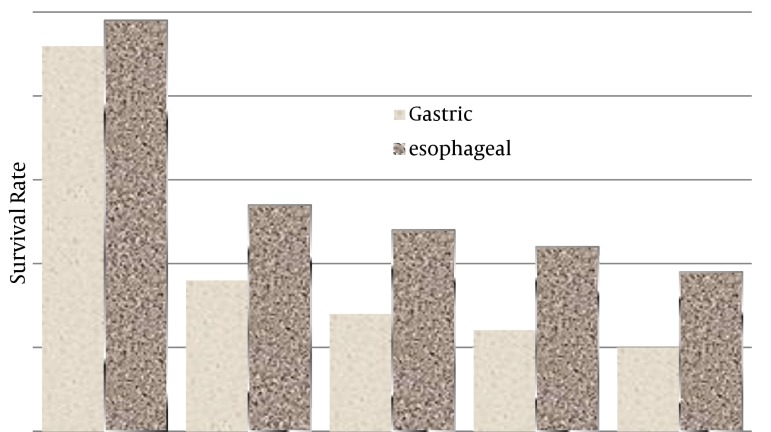
1 - 5 Year Survival Rates of Gastric and Esophageal Cancer

The five-year survival rates (by year) for esophageal cancer were 49%, 27%, 24%, 22% and 19% respectively and for gastric cancer were 41%, 17%, 13%, 10% and 5.4% respectively (Figure 2). 

**Figure 3.**
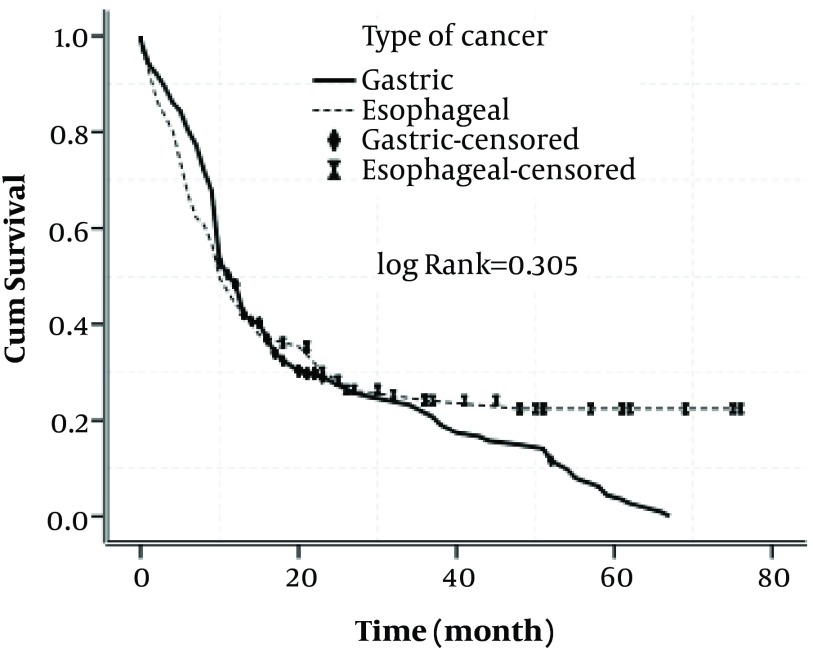
Figure 3. Cumulative Survival Rate in Patients With Gastric and Esophageal Cancers

**Table 5.** 1 - 5 Year Survival Rates of Gastric and Esophageal Cancer Related to Demographic and Histological Factors

Survival Cancer Variables	Survival rates, (%)					Med Tim Survival By Month
	1-year	2-year	3 -year	4-year	5-year	
Gastric						
Gender						
Male	45.1	18.2	13.5	12.6	7.3	11.56
Female	48.2	21.3	19.7	15.6	15.6	11.90
**Age**						
45 >	62.3	46.7	24.2	18.1	13.3	15.00
46-65	49.3	24.3	17.2	15.3	12.1	12.62
66 <	43.4	17.1	14.2	11.3	8.5	11.08
**Histological type**						
Intestinal	47.3	23.3	18.1	16.3	12.5	11.59
Diffuse	50.5	7.1	4.0	4.0	4.0	13.78
**Esophageal**						
**Gender**				19.5		
Male	46.2	23.3	19.5		13.7	11.27
Female	52.3	29.7	24.1	19.0	19.0	13.45
**Age**						
45 >	80.2	80.2	68.3	68.3	68.3	60.0
46 - 65	72.1	38.5	31.1	27.2	27.2	20.25
66 <	24.5	8.3	4.2	4.2	0.0	7.98
**Histological type**						
AC	21.2	19.2	5.1	1.2	0.0	7.67
SCC	56.2	32.1	25.5	22.0	19.0	15.43

## 5. Discussion

Geographic variability in prognosis of gastric cancer patients has been well documented ( 12 , 13 ). The minimum and maximum survival rates have been reported for European countries and East Asia, respectively ( 14 ). Similarly, the five-year survival rates for gastric cancer vary from 10% to 55% worldwide ( 15 ). In Iran, the prognosis of patients with gastric cancers remains poor ( 9 , 14 ). As a result both diseases are among the deadliest forms of cancer ( 16 ). In our study, the five-year overall survival rate for patients with esophageal cancer in this population (19%) is very poor and women had a better survival rate than men (0.345). This result agrees with a report from Europe ( 17 ). According to our data, there was a significant difference in survival rates between patients with SCC and those with AC of the esophagus. These results are in contrast with studies from the United Kingdom (1987 to 2000) ( 18 ) and Germany (1982 to 2000) ( 19 ), which showed that AC has a more favorable prognosis than SCC in esophageal cancer patients. In general, esophageal cancer five-year survival (19%) was poor, but it was slightly better than that of gastric cancer (5.4%) (P = 0.361). These figures are different from those found in countries such as England and South Korea ( 20 , 21 ). Major differences between these cancers were seen regarding survival rates of patients and their incidence classified by sex (Table 1). The present results showed that patients' gender had no significant impact on survival rate in both cancers. Median survival times of men and women for esophageal cancer were 10.0 ± 1.03 and 12.0 ± 2.83, respectively and for gastric cancer these were 11.0 ± 0.53 and 13.0 ± 0.94, respectively. Sex was not an independent prognostic factor in either Chinese patients (p = 0.23) ( 22 ) or white patients living in the United States (P = 0.28) ( 23 ). Overall survival was significantly worse only for male white patients compared with the Chinese patients (median survival time, 12.4 versus 14.5 months, respectively; P < .01) ( 22 ). However, some studies have found better survival rates for women ( 24 ). 

For esophageal cancer; patients under the age 45 had significantly better survival in univariate analysis. The reasons for this are likely to include a combination of better general health, more effective response to treatment and earlier diagnosis in younger people. Differences in underlying tumor biology may also play a part. A previous report indicated better survival for younger patients (22). On the other hand, some other studies have not reported the same (16). Cell histology is another tumor related factor that might affect patient survival. Our findings are in conformity with previous reports which have showed better survival for AC of esophagus (25). In this study, the Lauren classification (based on tumor histology) did not have a prognostic significance for five-year survival of gastric cancer patients for either population (log rank = 0.611). Treatment is likely to be the greatest determinant of cancer patients’ survival. Surgical results in treatment of esophageal cancer have improved significantly over recent years. Medical centers now report that patients undergoing surgery alone, have median survival rates between 13 and 19 months, 2-year survival rates between 35% and 42%, and 5-year survival rates of 15% to 24% (26). In the present study in patients for whom surgery was the only option for treatment, median of survival and 4-year survival rate of esophageal cancer was 12 ± 3.86 months and 21.3%, respectively (P = < 0.001). In patients for whom radiation therapy was the only option for treatment of esophageal and gastric cancer, the mean survival rate was 5 ± 1.06 months and 7 ± 1.29 months, respectively. Radiation therapy has been used in the past as a single-modality approach with a curative intent. However, except for those with very early-stage disease, radiation has had little impact on long-term survival (27). Chemotherapy is performed preoperatively, postoperatively, or both; median survival rates of chemotherapy were 5 ± 0.548 months for esophageal and 9 ± 1.28 months for gastric cancer. Multimodality treatment approaches have evolved over the recent years in response to the frequent loco regional and distant recurrences identified after surgery or radiation therapy alone.

The strength of the study was assured by the availability of homogeneous sample data with details of tumor histology and pathology, using Cox multiple regression analysis to adjust result variables on survival and calculation of five-year survival rates separately for sub-group analysis. There are limitations with the present study in which the survival rate was unable to predict future events for a particular person. Meanwhile, it was not possible to consider changes in characteristics after diagnosis, which may have affected survival. Hospital series often provide more optimistic data; there are challenges in interpreting registry information regarding the health care system of Iran. They are of limited value because of unavoidable selection bias, in particular in case selection and patient's characteristics. In conclusions, gastric and esophageal cancers are heterogeneous diseases, but they share important features. They remain clinically asymptomatic until late in the disease process with consequent poor prognoses and high mortality rates. This study points to differences in disease characteristics and patient factors. Even so, the outcomes of these cancers are poor and improvements in diagnosis and management are urgently needed.
